# Molecular Antimicrobial Resistance of* Neisseria gonorrhoeae* in a Moroccan Area

**DOI:** 10.1155/2018/7263849

**Published:** 2018-11-21

**Authors:** Safae Karim, Chahrazed Bouchikhi, Abdelaziz Banani, Hinde El Fatemi, Tiatou Souho, Sanaa Erraghay, Bahia Bennani

**Affiliations:** ^1^Laboratoire de Microbiologie et de Biologie Moléculaire, Faculté de Médecine et de Pharmacie de Fès (FMPF), Université Sidi Mohammed Ben Abdellah (USMBA), Fès, Morocco; ^2^Laboratoire de Biotechnologie, Faculté des Sciences Dhar El Mehraz, USMBA, Fès, Morocco; ^3^Service de Gynécologie CHU Hassan II de Fès, Morocco; ^4^Equipe Micro-Organismes, Génomique et Facteurs Oncogènes, Laboratoire de Pathologie humaine, biomédecine et Environnement, FMPF, USMBA, Fès, Morocco; ^5^Equipe Anatomo-Pathologie, Laboratoire Central d'Analyse Médicale, CHU Hassan II, Fès, Morocco

## Abstract

**Objectives:**

To identify the prevalence and the types of* Neisseria gonorrhoeae *(NG) resistance plasmids-mediated penicillin (PPNG) and tetracycline (TRNG), the ciprofloxacin resistance (CRNG), and related risk factors of each types of resistance.

**Methods:**

The beta-lactamase-producing plasmid types (Africa, Asia, and Toronto),* tetM *tetracycline resistance plasmid types (America and Dutch), and the determination of the Ser-91 mutation of GyrA were detected by specifics PCRs on 149 diagnosed NG positives samples followed by* Hinf1 *digestion for* tetM* and* gyrA *mutation.

**Results:**

135 (90.1%) samples showed a profile of molecular resistance to at least one antibiotic with predominance of ciprofloxacin resistance. In fact, 36 (24.2%) and 69 (46.3%) cases harbored PPNG and TRNG, respectively, and 116 (77.9%) cases showed the mutation Ser-91 of GyrA (CRNG). From a total of 36 PPNG isolates, the Toronto, Asian, and Toronto/Asian types were detected in 13 (36.1%), 10 (27.8%), and 13 (36.1%) cases, respectively, whereas the African type was not detected. In addition, the American type of TRNG was detected in 92.8% (64/69) of cases, while the Dutch type was detected in 7.2% (5/69) of cases. The association of demographics and clinical variables with NG resistance to ciprofloxacin, penicillin, and tetracycline was studied and the risk factors have been determined.

**Conclusion:**

Resistance to penicillin, tetracycline, and ciprofloxacin among NG samples positives remained at high levels in Morocco as determined by molecular profile. So, the use of molecular tools for NG antimicrobial resistance detection can help in the management and spread limitation of this infection.

## 1. Introduction

*Neisseria gonorrhoeae *(NG) is the second most bacterial sexually transmitted infection (STI) in the world [1]. It can infect the male and female genital tract and cause severe complications such as pelvic inflammatory diseases with possible sequelae in women, infertility in both genders, and neonatal conjunctivitis. Untreated gonorrhea can lead to inflammation of the mucous membranes and facilitates HIV transmission [2].

In 2012, WHO estimated that 78.3 million new gonorrhea cases occurred among women and men aged between 15 and 49 years [1]. Currently, there is no effective vaccine against gonococcal infections. However, infections and NG have developed antimicrobial resistance that has been used previously for its treatment, including to sulfonamides, penicillins, narrow-spectrum cephalosporins, tetracyclines, macrolides, and fluoroquinolones [3]. The plasmid-mediated penicillin-resistant NG (PPNG) has been largely described with high rates that may exceed 80% in some Asian countries [4]. This resistance is mostly associated with three types of *β*- lactamase producing plasmids: Asian, African, and Toronto types [5]. Also, plasmid-mediated tetracycline-resistant (TRNG) has been described with high rates that may exceed 70% in South Africa [6]. In fact, this resistance by TetM is carried by two conjugative plasmids (American and Dutch) of similar sizes [7], but with different* tetM* sequences and hence different profiles of endonuclease restriction fragments. However, the last years (2004-2012) were marked by high frequency of ciprofloxacin resistance that reached 99% [8]. This resistance is mainly related to* gyrA* and* parC *mutations with predominance of the mutation Ser-91 of GyrA [9]. Due to the emerging resistance of NG, Centers for Disease Control and Prevention have recommended several changes to the treatment guidelines (CDC). In 2012, the single-dose oral cephalosporin has been replaced by dual therapy: a single injection of ceftriaxone (250 mg) and single-dose azithromycin (1 g) administered orally.

In Morocco, a 2009 study conducted in men with urethral discharge showed high rates of penicillin, tetracycline, and ciprofloxacin NG resistance (56.2%, 92.6%, and 86.8%, respectively) [10]. On the basis of these results, the treatment of gonococcal infections was changed and the Ministry of Health replaced ciprofloxacin by single-dose ceftriaxone 250 mg [10]. However, this study was conducted on small sampling (76 cases) that is not representative of all Moroccan regions and in our knowledge, no study has been conducted to determine the mechanisms of antimicrobial resistance of NG circulating in Morocco.

The aims of this study were to identify the prevalence and the types of NG plasmids-mediated penicillinase-producing (PPNG) and Tetracycline-producing (TRNG) and the ciprofloxacin Ser-91 status in* gyrA *(CRNG) and to identify the associated risk factors.

## 2. Materials and Methods

### 2.1. Sampling

From 2013 to 2015, a prospective study was conducted among women consulting the Gynecology Department and the Anatomy-Pathology Laboratory of the Hassan II University Hospital of Fez to determine the prevalence and characterize bacterial STI notably NG. A total of 1053 patients aged 18 years and above, HIV negatives, not virgins, who not menses at the day of sampling and who accepted to provide their written consent were included in this previous study. From all collected cervical samples, 149 (14.1%) were tested positive for NG by PCR using specific primers [12] and are used in the present study to determine the molecular antimicrobial resistance of NG. The deidentified demographics and clinical data of participants are available. Only 31.5% (47/149) of those samples NG positives were obtained in symptomatic women (women presenting at least one of the following genital symptoms: leucorrhoea, pelvic pain/dyspareunia, pruritus, menorrhagia, metrorrhagia, or dysuria).

This study was approved by the Institutional Review Board of Fez, Morocco (N° 02/15).

### 2.2. Genotypic Characterization of NG

One hundred and forty-nine NG positives samples by PCR were used to determine the types of PPNG, TRNG, and CRNG

The detection of beta-lactamase-producing plasmid types (Africa, Asia, and Toronto),* tetM *tetracycline resistance plasmid types (America and Dutch), and the determination of the mutation Ser-91 in* gyrA *was performed by PCR using specific primers followed by* HinfI* digestion of* tetM* and* gyrA* products as previously described [9, 11]. Primers used, PCR amplicons sizes are listed in Table 1. Restriction fragments were determined in 1.5% agarose gel electrophoresis (Table 1). Positive and negative controls were used for each PCR.

### 2.3. Statistical Analysis

The statistical analysis was performed using SPSS (version 20, SPSS Inc., Chicago, Illinois, USA). Descriptive analysis was performed for all variables. The relationship between different variables and TRNG, PPNG, and ciprofloxacin resistant NG was checked using Fisher exact test or chi-squared test when applicable. Multivariate analysis was conducted using a binary logistic regression model to determine the risk factors. Variables included in the model were those with p-value ≤0.20 and the odds ratios (OR) and 95% confidence intervals (CI) were calculated. In all tests, the value p <0.05 was considered statistically significant.

## 3. Results

### 3.1. Studied Population

A total of 149 NG positives samples of consenting women were included in this study. The mean age of participants was 43.16 years (20-66 years) and 27 (18.1%) were pregnant. Illiteracy rate was 61.5%. Most of women were married (91.8%). Among them, 37.9% had more than three pregnancies and nearly 31% of them had more than three children. The characteristics of the included population are presented in Table 2.

### 3.2. Prevalence of TRNG, PPNG, and CRNG

In this study, samples containing at least one of the plasmids-encoding resistances for penicillin or tetracycline or the substitution in GyrA (Ser-91Phe) were considered resistant. Among the 149 NG positives samples, 135 (90.1%) showed a molecular resistance profile to at least one antibiotic with the predominance of resistance to ciprofloxacin. In fact, 36 (24.2%) NG cases harbored PPNG, 69 (46.3%) cases harbored TRNG, and 116 (77.9%) cases showed mutated Ser-91 in* gyrA*. The double resistance was detected in 50 cases (33.6%), while the triple resistance (TRNG/PPNG/CRNG) was found in 18 samples (12.1%) as reported in Table 3.

### 3.3. PPNG and TRNG Genotypes

From a total of 36 PPNG isolates, the Toronto, Asian, and Toronto/Asian types were detected in 13 (36.1%), 10 (27.8%), and 13 (36.1) cases, respectively, whereas the African type was not detected. In addition, the American type of TRNG was detected in 92.8% (64/69) of cases, while the Dutch type was detected in 7.2% (5/69) of cases. The genotypic profile of samples is presented in Table 3.

### 3.4. Association of Beta-Lactamase-Encoding and TetM-Encoding Plasmids

A total of 22 (61.1%) samples harboring PPNG plasmid also contained the TetM-encoding plasmid. This association is statistically significant (P=0.041). However, there was no significant association between the presence of beta-lactamase encoding plasmids and the type of TRNG variant plasmid (Table 4).

### 3.5. Prevalence of PPNG, TRNG, and CRNG according to Consultation Motifs

The association of NG resistance mechanisms with clinical symptoms was studied. The results show that highest percentages of PPNG and TRNG resistance (94.4% and 89.9%, respectively) were obtained in nonpregnant women than in pregnant women (5.6% and 10.1%, respectively) with P value <0.05) (Table 5). However, the TRNG is statistically associated with the absence of symptoms (p = 0.017) (Table 5).

### 3.6. Correlation between NG Resistance and Variables

The association of demographics and clinical variables with NG resistance profiles was studied in a univariate analysis. The results were reported in Tables 6 and 7.

All variables with p ≤ 0.20 were used in a step wise selection procedure in logistic regression analysis. In the final model, the obtained results show that literate women and those with high number of parity (>3) were at higher risk of TRNG (OR 95% IC1.623 [1.180–2.231]) and 2.920 [1.303–6.547], respectively). Those living in urban area and married ones seem to be at high risk of CRNG (p<0.05) compared to the others. However, literate patients were at higher risk of infection with species harboring two different PPNG and carrying triple drug resistance (PPNG/TRNG/CRNG) (Table 8).

## 4. Discussion

Gonococcal infection represents a major public health issue [13]. In fact, its extent of genetic recombination leads to a large phenotypic diversity that enhances the transmission and ability to escape the host immune systems [14]. This tendency for genetic transformation and recombination also leads to the rapid spread of antibiotics resistance genes in many countries of the world. This includes resistance to sulfonamides, macrolides, penicillins, tetracyclines, and fluoroquinolones [3]. These resistances are related in part to a Ser-91Phe substitution in* gyrA* for ciprofloxacin (Fluoroquinolones) resistant gonococcal strains, a* tetM *gene for plasmid-mediated tetracycline resistance (TRNG), and a penicillinase encoding plasmids (Asian, African, Toronto, Rio, Nîmes, New Zealand, and Johannesburg) for plasmid-mediated penicillin resistance (PPNG). However, the mutations in* folP *[encoding the sulfonamide target the enzyme dihydropteroate synthase (DHPS)] are linked to sulphonamide resistance and the* mtrR *mutations or* erm *genes to macrolides resistance [15].

In Morocco and especially in Fez regions area, the rate of NG infection recently detected based on molecular diagnosis was 14% (data not published). This rate is considered very high compared to the prevalence noted in the African regions (2.3%) [16]. Despite this, there is no information on the extent and mechanisms of gonorrhea resistance from this area.

This study provides the first data on PPNG, TRNG, and* gyrA* status in Morocco and helps to estimate the NG resistance rates. In fact, PPNG was firstly reported in United Kingdom and Thailand in 1976 [17] and TRNG was initially found in the United States in 1985 [18] and their frequency has increased over time.

In our study, PPNG was identified in 24.2% NG positive samples. This rate is similar to that detected in South Africa (25.8%) in 2010 [6], but higher than the rates observed in Rio de Janeiro (8.6%) [19] and in Manaus (14.5%) [20]. However, it still lower than that found in some Asian countries like Indonesia (60% from Bandung and 62.1% from Jakarta in 2003) [21], Bhutan (86% from 2007 to 2011) [4], and Thailand (82.1% in 2012) [22]. Comparisons between these results and the study results must be interpreted with caution since the epidemiology of resistant isolates differs in the time and the detected rates were determined in different years and in populations with different risk factors.

The PPNG were genotyped and the results showed that Toronto and Asian types were detected separately or in combination, whereas the African type was not detected. Unfortunately, data on PPNG types circulating in neighboring North African countries are not available and the only African data that exists are those reported in 2010 from South Africa who showed different distribution types with predominance of Toronto (44.4%) and African (35.2%) types and absence of Asian type [6]. However, during the same period (2010), the African type was the most prevalent in some Asian countries like Bangkok, Thailand, Japan, and Bangladesh [22–24]. Even if resistance profiles distribution can change over the time, the present results let us suppose that the circulating isolates, in a given period of time, are different according to geographical area.

Samples with two PPNG types (Toronto/Asian) were present with high frequency (36.1%). This can be explained by the fact that the infecting strains harbor two plasmids or by mixed gonococcal infection. This last hypothesis seems more plausible since (1) the presence of two plasmids with the same origin of replication is unusual and (2) mixed gonococcal infection at the same anatomical site has been documented and proven by culture in several studies [25–28]. In fact, those studies reported different rates of mixed infections according to studied population (1.3%-3.2% in Australia and 40% in sex worker, USA). The results of the present study give an evidence of the high rate of mixed infection and show that PPNG typing can be helpful in their detection. It also shows the advantage of molecular techniques used directly on clinical samples, for epidemiological antimicrobial resistance (AMR) surveillance.

The NG mixed infections can be explained by sexual behaviors of patients and/or their partners. In fact, individuals with multiple sexual partners are at a higher risk of mixed NG infections and risk is higher in areas with a high incidence of gonococcal infections. In our study, the majority of women stated that they only had one partner. In this case, simultaneous transmission of mixed strains by one partner that has been documented by Matin and Ison [27] can explain the mixed infection. To verify this hypothesis, information about sexual behavior would be required from the partner and testing of the partner should be done.

The prevalence of TRNG strains (49.5%) is similar to that found in China (52.1%) in 2015 [29] and higher than those observed in some South American countries: Manaus (12.7%) [20] and Rio de Janeiro (20%) [19]. However it still lower than the rates reported in South Africa (70.3%) [6] and in Thailand (84.1%) [22] in 2010 and 2012, respectively.

In our study, we found that American type TRNG is predominant with rate of 92.8% and the Dutch type is present only in 7.2% of cases. This* tetM* plasmids types distribution is similar to that obtained in Africa [98.3% (59/60) American and 0.7% (1/60) Dutch type] [30], in South Africa [76% (117/154) and American type 24% (37/154) Dutch] [6], and in Europe [82% (248/306) American and 18% (58/306) Dutch type] [30]. However, it is different from that observed in North, Central, and South America [40% (4/10) American and 60% (6/10) Dutch type] [30], in Asia [100% (35/35) Dutch type; 98.7% (377/382) Dutch and 1.3% (5/382) American type], in Bangladesh [30, 31], and in China [100% (639/639) Dutch type] [29]. These findings confirmed that the American type was the most prevalent in Africa including Morocco.

Our findings show also that the most PPNG positive samples 22 (61.1%) carried also the TetM-encoding plasmid. This is in agreement with the hypothesis supposing that the* tetM* plasmids plays a role in the mobilization of beta-lactamase plasmids [32]. Potentially the American type* tetM* plasmid is effective at mobilizing the Toronto type plasmids, as proved by the high frequency of this type of penicillinase-producing plasmid in samples containing the American type* tetM* plasmid.

Previous studies have demonstrated that Ser-91 mutation in* gyrA* for NG is mainly associated with ciprofloxacin resistance [9]. Even if other mutations have been reported in ciprofloxacin resistant isolates, several studies have proved that they usually occur in parallel with* gyrA* mutation. Thus, the GyrA status can be used alone for ciprofloxacin susceptibility prediction [33, 34]. In our study, a total of 116 (77.9%, 116/149) isolates showed the mutation Ser-91 in* gyrA *extrapolating to ciprofloxacin resistance. However, high level of CRNG has been worldwide reported and especially in Asia (93.8% in 2003; 100% from Hefei) [8, 35].

The results of this study, not previously done on Moroccan samples, add to our epidemiological knowledge and reinforces that these antibiotics (penicillin, tetracycline, and ciprofloxacin) should not be used for STI management and especially for NG, in Morocco. The results reinforce the treatment change made by the Moroccan Ministry of Health [10].

In our study, we have found that the higher percentage of PPNG and TRNG resistance were detected in nonpregnant women compared to pregnant women. Also, the higher level of TRNG was associated with asymptomatic women for genital STIs. The fact that NG causes only mildly symptomatic or asymptomatic infections, especially in women, increases the probability of disease progression and TRNG resistant isolates transmission [36] especially when the diagnosis is not routinely performed. Therefore, it is necessary to make a systematic screening for this infection.

The determination of risk factors may help in patient management and future prevention strategies. In our study, literate patients and those with high parity have a significant 2.8-fold (95%, CI, 1.295–6.045) and 2.9-fold (95%, CI, 1.303–6.547), respectively, increased risk of TRNG in comparison with illiterate women and those with ≤3 parities. Besides, there was a higher risk of infection with CRNG for married women living in urban areas than it for divorced or widowed living in rural area. However, the risk of being infected by two PPNG plasmids types and possessing the triple drug resistance was 4.5-fold (95%, CI, 1.059–19.407) and 2.8-fold (95%, CI, 1.041–7.907), respectively, higher in literate patients than in illiterate women.

Those results indicate that literate women, married women, those living in urban area, and those with high number of parity seem to be at higher risk to be infected with resistant NG. The risk determined for literate women and those living in urban area can be explained by (i) self-medication (more easily and widely used by literate people), leading to the misuse of antibiotics and therefore the resistance development; (ii) easy access to pharmacies and antibiotics (without a medical prescription) in urban area.

The risk determined for married patients and those with high parity can be explained by the fact that those women are usually sexually active and the probability of invasive acts related to delivery.

Further studies are needed to understand the influence of behavioral and socioeconomic factors on the resistance of NG to specific antibiotics such as tetracycline, penicillin, and ciprofloxacin.

This study has some limitations. In fact, low number of samples was used and only few genes were targeted to determine the resistance rates in the absence of phenotypic characterization. Nevertheless, having an estimation of NG resistance is useful for treatment strategy, especially in region where bacterial culture and characterization are not performed and patients are generally not diagnosed for STIs.

## 5. Conclusion

In summary, this study highlights the high rate of NG Moroccan isolates resistance to antimicrobials previously recommended for the treatment of gonorrhea (penicillin, tetracycline, and ciprofloxacin). The high rate of mixed infections shows the failure of NG and STI management. Thus, the use of molecular tools for STIs diagnosis and antimicrobial resistance detection can help in their management.

## Figures and Tables

**Table 1:** Primer sequences, targeted genes, and PCR amplicons sizes.

**Primers**	**Sequence (5' → 3')**	**Targeted gene**	**Type**	**PCR amolicons sizes (bp) /*HinfI***		**Reference**
				Before digestion	After digestion	
**GC1F**	AACTCACGGACAAAATCACGG	*β*-lactamase producing Plasmid	Africa	1070	NA	[11]
**GC2F**	CACCTATAAATCTCGCAAGCC		Asia	737	NA	
**GC3R**	AACGCAAGCAGGACGAAATC		Toronto	435	NA	
**GC4R**	CCTCCACCTTCATCCTCAGC					
**TetMF**	ACTGTTGAACCGAGYAAACCT	*tetM*	American	841	748+93	
**TetMR**	TCTATCCGACTATTTGGACGACG		Dutch	841	572+176+93	

**GyrAF**	CGGCGCGTACTGTACGCGATGCA	Gyrase A	Mutation Ser-91	278	278	[9]
**GyrAR**	ATGTCTGCCAGCATTTCATGTGAGA		Wild type	278	166+112	

NA: not applicable.

**Table 2:** Demographics and clinical characteristics of the studied population.

**Variable**		**N**	%
**Age group (years), n=148**	≤42	65	43.9
	>42	83	56.1

**Education level, n=148**	Illiterate	91	61.5
	Primary school	24	16.2
	Secondary school	17	11.5
	College	16	10.8

**Family status, n=147**	Married	135	91.8
	Divorced/Widowed	12	8.2

**Number of pregnancies, n=145**	≤3	90	62.1
	>3	55	37.9

**Parity, n=145**	≤3	100	69.0
	>3	45	31.0

**History of miscarriages, n=144**	No	103	71.5
	Yes	41	28.5

**Menopause, n=145**	No	91	62.8
	Yes	54	37.2

**History of lesions, **n**=145**	No	113	77.9
	Yes	32	22.1

**Sampling department, **n**=149**	Gynecology department	59	39.6
	Laboratory of Anatomopathology	90	60.4

**Number of lifetime sexual partners, n = 145**	1	137	94.5
	≥2	8	5.5

**Passive smoking, n = 144**	No	109	75.7
	Yes	35	24.3

**Oral contraception, n = 146**	No	103	70.5
	Yes	43	29.5

**Age at 1st sexual intercourse (years), n = 147**	≤21	98	66.7
	>21	49	33.3

**Prenatal consultation**	No	122	81.9
	Yes	27	18.1

**Genital symptoms**	No	102	68.5
	Yes	47	31.5

**Infertility**	No	142	95.3
	Yes	7	4.7

**Table 3:** Genotypic characterization of 145 NG positive samples.

	**Mono drug resistance**			**Double drug resistance**			**Triple drug resistance**
	**PPNG**	**TRNG**	**CRNG**	**PPNG/TRNG**	**PPNG/** **CRNG**	**TRNG/CRNG**	**PPNG/TRNG/CRNG**

**PPNG**							
Africa	-	-	-	-	-	-	-
Asia	-	-	-	2	3	-	5
Toronto	1	-	-	2	7	-	3
Toronto/Asia	-	-	-	-	3	-	10
Total	**1**	-	-	**4**	**13**	-	**18**
**TRNG**							
America	-	13	-	4	-	30	17
Dutch	-	1	-	-	-	3	1
Total	-	**14**	-	**4**	-	**33**	**18**
**CRNG**							
Mutation S91 GyrA	-	-	**52**	-	**13**	**33**	**18**

PPNG, penicillinase-producing *N. gonorrhoeae *(plasmid-mediated); TRNG, tetracycline resistant *N. gonorrhoeae *(plasmid-mediated); CRNG: ciprofloxacin-resistant *N. gonorrhoeae.*

**Table 4:** Distribution of the beta-lactamase-encoding and TetM-encoding plasmids among 149 NG samples positives.

	**PPNG**				**Not PPNG**		
	Asia plasmid	Toronto plasmid	Asia/Toronto plasmid	Total			
**TRNG category**	N (%)					P-value	
TetM encoding American plasmid	5 (38.5)	7 (70.0)	9 (69.2)	21 (58.3)	43 (38.1)	0.486	0.100
TetM encoding Dutch Plasmid	0 (0.0)	0 (0.0)	1 (7.7)	1 (2.8)	4 (3.5)		
**No TetM encoding plasmid **	8 (61.5)	3 (30.0)	3 (23.1)	14 (38.9)	66 (58.4)	**0.041**	
**TetM encoding plasmid**	5 (38.5)	7 (70.0)	10 (76.9)	22 (61.1)	47 (41.6)		

PPNG, penicillinase-producing *N. gonorrhoeae *(plasmid-mediated); TRNG, tetracycline resistant *N. gonorrhoeae *(plasmid-mediated).

**Table 5:** Prevalence of PPNG, TRNG, and CRNG according to consultation motifs.

**Reason for visiting**		**PPNG+**		**TRNG+**		**CRNG+**	
		N (%)	P-value	N (%)	P-value	N (%)	P-value
**Prenatal consultation **	No	34(94.4)	**0.025**	62(89.9)	**0.019**	94(81.0)	0.616
	Yes	2(5.6)		7(10.1)		22(19.0)	

**Genital symptoms**	No	28(77.8)	0.167	54(78.3)	**0.017**	78(67.2)	0.550
	Yes	8(22.2)		15(21.7)		38(32.8)	

**Infertility**	No	35(97.2)	0.462	65(94.2)	0.484	111(95.7)	0.484
	Yes	1(2.8)		4(5.8)		5(4.3)	

PPNG, penicillinase-producing *N. gonorrhoeae *(plasmid-mediated); TRNG, tetracycline resistant *N. gonorrhoeae *(plasmid-mediated); CRNG: ciprofloxacin-resistant *N. gonorrhoeae*.

**Table 6:** Association between global resistance, TRNG, PPNG, CRNG, and variables.

**Variable**		**Global resistance, N (**%**)**		**TRNG, N (**%**)**		**PPNG, N (**%**)**		**CRNG, N (**%**)**	
		**Yes**	**No**	**Yes**	**No**	**Yes**	**No**	**Yes**	**No**
**Age group (years)**	≤42	58(89.2)	7 (10.8)	26(40.0)	39 (60.0)	12(18.5)	53 (81.)	52(80.0)	13 (20.0)
	>42	76(91.6)	7 (8.4)	43(51.8)	40 (48.2)	24 (28.9)	59 (71.1)	63 (75.9)	20 (24.1)
P-value		0.630		0.153		0.141		0.552	

**Living area**	Rural	23(82.1)	5 (17.9)	13(46.4)	15 (53.6)	7 (25.0)	21 (75.0)	17 (60.7)	11 (39.3)
	Urban	111(92.5)	9 (7.5)	56 (46.7)	64 (53.3)	29 (24.2)	91 (75.8)	98 (81.7)	22 (18.3)
P-value		0.097		0.982		0.926		**0.016**	

**Family status**	Married	122(90.4)	13 (9.6)	63 (46.7)	72 (53.3)	33 (24.4)	102(75.6)	108 (80.0)	27 (20.0)
	Widowed /divorced	11(91.7)	1 (8.3)	6 (50.0)	6 (50.0)	3 (25.0)	9 (75.0)	6 (50.0)	6 (50.0)
P-value		0.680		0.825		0.601		**0.027**	

**Education level**	Illiterate	83(91.2)	8 (8.8)	38 (41.8)	53 (58.2)	22 (24.2)	69 (75.8)	67 (73.6)	24 (26.4)
	Literate	51(89.5)	6 (10.5)	31 (54.4)	26 (45.6)	14 (24.6)	43 (75.4)	48 (84.2)	9 (15.8)
P-value		0.726		0.134		0.985		0.132	

**Number of pregnancy**	≤3	80(88.9)	10 (11.1)	37 (41.1)	53 (58.9)	21 (23.3)	69 (76.7)	74 (82.2)	16 (17.8)
	>3	52(94.5)	3 (5.5)	31 (56.4)	24 (43.6)	15 (27.3)	40 (72.7)	40 (72.7)	15 (27.3)
P-value		0.198		0.074		0.594		0.176	

**Parity**	≤3	90(90)	10 (10)	42 (42.0)	58 (58.0)	23 (23.0)	77 (77.0)	83(83.0)	17 (17.0)
	>3	42(93.3)	3 (6.7)	26 (57.8)	19 (42.2)	13 (28.9)	32 (71.1)	31(68.9)	14 (31.1)
P-value		0.381		0.078		0.448		0.055	

**History of miscarriage**	No	91(88.3)	12 (11.7)	43 (41.7)	60 (58.3)	23 (22.3)	80 (77.7)	81 (78.6)	22 (21.4)
	Yes	39(95.1)	2 (4.9)	23 (56.1)	18 (43.9)	12 (29.3)	29 (70.7)	31 (75.6)	10 (24.4)
P-value		0.179		0.119		0.381		0.693	

**Menopause**	No	83(91.2)	8 (8.8)	44 (48.4)	47 (51.6)	21 (23.1)	70 (76.9)	74 (81.3)	17 (18.7)
	Yes	48(88.9)	1 (11.1)	24 (44.4)	30 (55.6)	15 (27.8)	39 (72.2)	39 (72.2)	15 (27.8)
P-value		0.647		0.649		0.526		0.202	

**Passive smoking**	No	98(89.9)	11 (10.1)	49 (45.0)	60 (55.0)	25 (22.9)	84 (77.1)	86 (78.9)	23 (21.1)
	Yes	32(91.4)	3 (8.6)	19 (54.3)	16 (45.7)	11 (31.4)	24 (68.6)	26 (74.3)	9 (25.7)
P-value		0.544		0.336		0.313		0.568	

**Use of oral ** **contraception**	No	95 (92.2)	8 (7.8)	51 (49.5)	52 (50.5)	27 (26.2)	76 (73.8)	78 (75.7)	25 (24.3)
	Yes	37 (86)	6 (14)	17 (39.5)	26 (60.5)	9 (20.9)	34 (79.1)	36 (83.7)	7 (16.3)
P-value		0.195		0.271		0.5		0.287	

**Age at first sexual intercourse ** **(years)**	≤21	90 (91.8)	8 (8.2)	46 (46.9)	52 (53.1)	25 (25.5)	73 (74.5)	76 (77.6)	22 (22.4)
	>21	43 (87.8)	6 (12.2)	23 (46.9)	26 (53.1)	11 (22.4)	38 (77.6)	38 (77.6)	11 (22.4)
P-value		0.303		1		0.684		1	

PPNG, penicillinase-producing *N. gonorrhoeae *(plasmid-mediated); TRNG, tetracycline resistant *N. gonorrhoeae *(plasmid-mediated); CRNG: ciprofloxacin-resistant *N. gonorrhoeae.*

**Table 7:** Association between PPNG type, double resistance, triple drug resistance, and variables.

**Variable**		**PPNG type, N (%)**		**Double resistance, N (%)**		**Triple resistance, N (%)**	
						**(PPNG/TRNG/CRNG)**	
		**Yes**	**No**	**Yes**	**No**	**Yes**	**No**
**Age group (years)**	≤42	7 (58.3)	5 (41.7)	20 (30.8)	45 (69.2)	6 (9.2)	59 (90.8)
	>42	16 (66.7)	8 (33.3)	30 (36.1)	53 (63.9)	12 (14.5)	71 (85.5)
P-value		0.447		0.493		0.334	

**Living area**	Rural	5 (71.4)	2 (28.6)	8 (28.6)	20 (71.4)	3 (10.7)	25 (89.3)
	Urban	18 (62.1)	11 (37.9)	42 (35.0)	78 (65.0)	15 (12.5)	105 (87.5)
P-value		0.501		0.517		0.795	

**Family status**	Married	21 (63.6)	12 (36.4)	48 (35.6)	87 (64.4)	17 (12.6)	118 (87.4)
	Widowed/divorced	2 (66.7)	1 (33.3)	2 (16.7)	10 (83.3)	1 (8.3)	11 (91.7)
P-value		0.709		0.157		0.666	

**Education level**	Illiterate	17 (77.3)	5 (22.7)	30 (33.0)	61 (67.0)	7 (7.7)	84 (92.3)
	Literate	6 (42.9)	8 (57.1)	20 (35.1)	37 (64.9)	11 (19.3)	46 (80.7)
P-value		**0.036**		0.791		**0.036**	

**Number of pregnancy**	≤3	11 (52.4)	10 (47.6)	28 (31.1)	62 (68.9)	12 (13.3)	78 (86.7)
	>3	12 (80)	3 (20)	22 (40)	33 (60)	6 (10.9)	49 (89.1)
P-value		0.089		0.275		0.668	

**Parity**	≤3	13 (56.5)	10 (43.5)	34 (68.0)	66 (66.0)	12 (12.0)	88 (88.0)
	>3	10 (76.9)	3 (23.1)	16 (32.0)	29 (64.4)	6 (13.3)	39 (86.7)
P-value		0.195		0.855		0.822	

**History of miscarriage**	No	16 (69.6)	7 (30.4)	32 (31.1)	71 (68.9)	12 (11.7)	91 (88.3)
	Yes	7 (58.3)	5 (41.7)	17 (41.5)	24 (58.5)	5 (12.2)	36 (87.8)
P-value		0.382		0.235		0.564	

**Menopause**	No	11 (52.4)	10 (47.6)	32 (35.2)	59 (64.8)	12 (13.2)	79 (86.8)
	Yes	12 (80)	3 (20)	18 (33.3)	36 (66.7)	6 (11.1)	48 (88.9)
P-value		0.089		0.823		0.714	

**Passive smoking**	No	17 (68)	8 (32)	40 (36.7)	69 (63.3)	11 (10.1)	98 (89.9)
	Yes	6 (54.5)	5 (45.5)	10 (28.6)	25 (71.4)	7 (20.0)	28 (80.0)
P-value		0.342		0.380		0.109	

**Use of oral contraception**	No	19 (70.4)	8 (29.6)	33 (32.0)	70 (68.0)	14 (13.6)	89 (86.4)
	Yes	4 (44.4)	5 (55.6)	17 (39.5)	26 (60.5)	4 (9.3)	39 (90.7)
P-value		0.158		0.384		0.472	

**Age at first sexual intercourse (years)**	≤21	18 (72)	7 (28)	31 (31.6)	67 (68.4)	13 (13.3)	85 (86.7)
	>21	5 (45.5)	6 (54.5)	19 (38.8)	30 (61.2)	5 (10.2)	44 (89.8)
P-value		0.126		0.389		0.594	

PPNG, penicillinase-producing *N. gonorrhoeae *(plasmid-mediated); TRNG, tetracycline resistant *N. gonorrhoeae *(plasmid-mediated); CRNG: ciprofloxacin-resistant *N. gonorrhoeae*.

**Table 8:** Variables associated with NG resistance types (multivariate analysis).

	**Variables**		**Odds ratio**	**95**%** CI**	**P-value**
**TRNG**	**Education level**	Illiterate	1		
		Literate	2.798	1.295 – 6.045	**0.009**
	**Parity**	≤3	1		
		>3	2.920	1.303 – 6.547	**0.009**

**CRNG**	**Area**	Rural	1		
		Urban	3.680	1.458-9.290	**0.006**
	**Family status**	Married	1		
		Widowed/divorced	0.176	0.050-0.614	**0.006**

**PPNG (double)**	**Education level**	Illiterate	1		
		Literate	4.533	1.059 – 19.407	**0.042**

**Triple drug resistance**	**Education level**	Illiterate	1		
**(PPNG/TRNG/CRNG)**					
		Literate	2.870	1.041 – 7.907	**0.041**

PPNG, penicillinase-producing *N. gonorrhoeae *(plasmid-mediated); TRNG, tetracycline resistant *N. gonorrhoeae *(plasmid-mediated); CRNG: ciprofloxacin-resistant *N. gonorrhoeae.*

## Data Availability

The data used to support the findings of this study are available from the corresponding author upon request.
